# Modeling Novel Putative Drugs and Vaccine Candidates against Tick-Borne Pathogens: A Subtractive Proteomics Approach

**DOI:** 10.3390/vetsci7030129

**Published:** 2020-09-07

**Authors:** Abid Ali, Shabir Ahmad, Abdul Wadood, Ashfaq U. Rehman, Hafsa Zahid, Muhammad Qayash Khan, Javed Nawab, Zia Ur Rahman, Abdulaziz S. Alouffi

**Affiliations:** 1 Department of Zoology, Abdul Wali Khan University Mardan, Khyber Pakhtunkhwa 23200, Pakistan; uop_ali@yahoo.com (A.A.); shabirjan427@gmail.com (S.A.); hafsa.zahid@awkum.edu.pk (H.Z.); qayashkhan@awkum.edu.pk (M.Q.K.); 2 Department of Biochemistry, Abdul Wali Khan University Mardan, Khyber Pakhtunkhwa 23200, Pakistan; awadood@awkum.edu.pk (A.W.); serendifity_rehman@outlook.com (A.U.R.); 3 State Key Laboratory of Microbial Metabolism, Department of Bioinformatics and Biostatistics, National Experimental Teaching Center for Life Sciences and Biotechnology, College of Life Sciences and Biotechnology, Shanghai Jiao Tong University, Shanghai 200240, China; 4 Department of Environmental Sciences, Abdul Wali Khan University Mardan, Khyber Pakhtunkhwa 23200, Pakistan; javednawab11@yahoo.com; 5 Department of Microbiology, Abdul Wali Khan University Mardan, Khyber Pakhtunkhwa 23200, Pakistan; zrahman@awkum.edu.pk; 6 King Abdulaziz City for Science and Technology, Riyadh 12354, Saudi Arabia

**Keywords:** tick, tick-borne pathogens, subtractive proteome analysis, homology modeling, MD simulation

## Abstract

Ticks and tick-borne pathogens (TBPs) continuously causing substantial losses to the public and veterinary health sectors. The identification of putative drug targets and vaccine candidates is crucial to control TBPs. No information has been recorded on designing novel drug targets and vaccine candidates based on proteins. Subtractive proteomics is an in silico approach that utilizes extensive screening for the identification of novel drug targets or vaccine candidates based on the determination of potential target proteins available in a pathogen proteome that may be used effectively to control diseases caused by these infectious agents. The present study aimed to investigate novel drug targets and vaccine candidates by utilizing subtractive proteomics to scan the available proteomes of TBPs and predict essential and non-host homologous proteins required for the survival of these diseases causing agents. Subtractive proteome analysis revealed a list of fifteen essential, non-host homologous, and unique metabolic proteins in the complete proteome of selected pathogens. Among these therapeutic target proteins, three were excluded due to the presence in host gut metagenome, eleven were found to be highly potential drug targets, while only one was found as a potential vaccine candidate against TBPs. The present study may provide a foundation to design potential drug targets and vaccine candidates for the effective control of infections caused by TBPs.

## 1. Introduction

Ticks are ectoparasites and notorious vectors for disease-causing pathogens that transmit various arboviruses, bacteria, and protozoans to vertebrate hosts adversely affecting the livestock industry and public health [1,2,3,4]. Some of the tick-borne pathogens (TBPs), such as bacteria (*Rickettsia rickettsii, Francisella tularensis*, *Ehrlichia chaffeensis, Anaplasma phagocytophilum, Borrelia burgdorferi*), protozoans (*Babesia* spp., *Theileria* spp.), and viruses (Crimean–Congo hemorrhagic fever virus, tick-borne encephalitis virus), cause a variety of diseases in infected hosts [5,6,7,8,9,10,11]. Human and animal movements associated with environmental changes have favored the dispersal of ticks and TBPs [12,13]. Therefore, the emergence and re-emergence of several TBPs pose public and veterinary health risks. For instance, tick-borne diseases, such as borreliosis, ehrlichiosis, anaplasmosis, and rickettsiosis, are some of the diseases emerging in regions where they have not been reported previously [14,15,16,17].

Recent progress in the field of bioinformatics has generated various in silico strategies and drug designing approaches that reduce the time and cost associated with the trial and error experimentations for drug development [18,19]. These methods serve to shortlist the potential drug targets that may be used for experimental validation. Subtractive proteomics is an in silico method used for the identification of essential and non-host homologous proteins within a pathogen proteome [18,20,21]. By selecting essential proteins unique to pathogen survival and propagation, the subtractive proteomics approach allows the identification of novel drug targets within a pathogen. The Database of Essential Gene (DEG) server can be used for the identification of those proteins involved in central metabolic pathways required for the survival of a pathogen.

The identification of proteins homologous to the proteins in the host gut can be screened out during the prediction of computer-based drug targets or vaccine candidates to avoid potential adverse effects of a drug. Target proteins selected through this approach may be used as a promising tool to control the diseases caused by infectious agents [22]. Subtractive proteome analysis has already been utilized for the identification of novel drug targets and vaccine candidates against several life-threatening pathogens such as *Pseudomonas aeruginosa* [23], *Streptococcus pneumonia* [24], and *Mycobacterium tuberculosis* [25,26].

Vaccination is a promising and sustainable approach to controlling ticks and TBPs [27,28]. Various in silico and drug design approaches have generated a plethora of data by eliminating the time and cost involved in trial and error experimentations during a drug or vaccine development [18,29,30,31,32,33,34]. Inceptive steps in the discovery of a novel drug target or vaccine candidates include the identification of target proteins [35]. To the best of our knowledge, limited studies have been reported using subtractive proteome analysis for the identification of drug targets or vaccine candidates against TBPs such as *B. burgdorferi* ZS7 [36] and *Rickettsia rickettsii* [37]. The purpose of this study is an in silico approach using subtractive proteomics for the prediction of potential drug targets and vaccine candidates against TBPs.

## 2. Methodology

### 2.1. Retrieval of Pathogens Proteome

In this study, TBPs were selected which had not been previously reported in similar in silico studies, had available complete proteome in the National Center for Biotechnology Information (NCBI), their name availability in the KEGG (Kyoto Encyclopedia of Genes and Genomes pathway database), their KO (KEGG Orthology) list provided by KAAS (KEGG Automatic Annotation Server), and their KO list of proteins available in KEGG pathways. The analysis of other TBPs was excluded in this study due to the fact of their available published reports and unavailability of their complete proteome and KO number in the KEGG database. The complete proteomes of selected pathogens, including *Borrelia burgdorferi* B31, *Ehrlichia chaffeensis* str. Arkansas, *Rickettsia rickettsii* str. “Sheila Smith”, *Francisella tularensis* SCHU S4, and *Anaplasma phagocytophilum* HZ, were retrieved in Fast Adaptive Shrinkage Threshold Algorithm (FASTA) format from NCBI.

### 2.2. Identification of Essential and Non-Host Homologous Proteins in Pathogens

To identify paralogous, duplicate or redundant sequences (when one or more homologous sequences are present in the same set of data) [38,39], the proteome of each pathogen was subjected to CD-HIT (cluster database at high identity with tolerance) with a sequence identity cut-off value of 0.4 (40%) [40,41]. Those proteins having more than a 40% identity were considered as paralogs in this analysis. The paralog protein sequences were excluded, and the non-paralog protein sets were subjected to the Basic Local Alignment Search Tool (BLASTp) at NCBI [42] against the host (*Homo sapiens* and *Bos taurus*) with threshold expected value (E-value) 10^−5^ to identify the non-host homologous proteins in pathogens. To screen the essential proteins, the retrieved non-homologous protein sequences, which were not present in the host (*H. sapiens* and *B. taurus*), were subjected to BLASTp against DEG to obtain essential genes [43,44]. The cut-off for E-value, bit score, and percentage of identity were considered <E 10^−10^, ≥100, and >35%, respectively [43,45,46]. A minimum bit score of 100 was used to screen out proteins that represented essential genes. The resultant data set revealed the non-homologous essential proteins of pathogens.

### 2.3. Metabolic Pathways and Subcellular Localization Analysis

The pathogen-specific metabolic pathways were predicted by subjecting the non-homologous proteins to KAAS and KEGG [47,48,49,50]. The proteins were separated based on their role in pathogen-specific unique metabolic pathways. The online server subCELlular Localization (CELLO) V.2.5 [51] was used for the prediction of subcellular localization of these proteins.

### 2.4. Druggability, Virulency Antigenicity, and Allergenicity Analysis

The vital non-host homologous proteins of pathogens were BLASTp against the DrugBank database which contains the Food and Drug Administration (FDA) approved drugs. As previously reported [52], target proteins with a bit score > 100, E-value 10^−5^, and having more than 50% identity with the drug targets present in the DrugBank database were selected as druggable. Virulence factors (VFs) of selected pathogen proteins were identified by performing BLASTp searches against the Virulence Factors Database (VFDB) core data set (R1) with a cut-off bit score > 100, and the E-value was 10^−5^ [53]. Vaxijen, an antigen alignment independent prediction tool was used for antigenicity analysis, and the AllerTOP v.2.0 server was used to predict allergenicity. The predicted antigenic score for each protein was categorized into a high antigenic and non-antigenic score. Proteins having antigenic scores more than 0.4 (default threshold value 40%) were considered highly antigenic, whereas those having less than 0.4 scores were considered as non-antigenic. Proteins with a high antigenic score were selected, and the NetCTL 1.2 server [54] was used for the prediction of potential T-cell epitopes. The Immune Epitopes Database was used to find the interaction between the T-cell epitope and MHC-I molecule (IEDB). To predict B-cell epitopes, a set of bioinformatics tools was used including the Kolaskar and Tongaonkar antigenicity scale [55], Emini surface accessibility prediction [56], Karplus and Schulz flexibility prediction [57], Bepipred linear epitope prediction analysis [54], and Chou and Fasman β-turn prediction analysis [58,59]. ProtParam [60] predicted the molecular weight, instability index, approximate half-life, isoelectric pH, GRAVY values, hydropathicity, and aliphatic index of the vaccine candidates.

### 2.5. Human Gut-Metagenomes Screening and Secondary Structure Prediction

To knock out pathogen proteins found in human gut flora, essential, non-homologous, and virulent proteins of *B. burgdorferi* B31, *E. chaffeensis* str. Arkansas, *F. tularensis* SCHU S4, and *A. phagocytophilum* HZ were scanned by BLASTp with an E-value cut-off score of 1 against proteins of the human gut flora using Human Microbiome Project database server [61].

The self-optimized prediction method by SOPMA alignment software [62] and Position-Specific Iterative Basic Local Alignment Search Tool (PSI-BLAST) based secondary structure prediction (PSIPRED) program were used to predict the secondary structure of the target proteins.

### 2.6. Phylogenetic Analysis

The amino acid sequence of the vaccine candidate (*B. burgdorferi* B31 FLiS protein) identified in this study was scanned for homologous sequences by BLASTp at NCBI. The homologous sequences were downloaded in FASTA format and were aligned using ClustalW in BioEdit Sequence Alignment Editor v.7.0.5 [63]. The evolutionary relationship of sequences was constructed using the neighbor-joining method in MEGA v. X [64] with bootstrapping at 1000 replications [65].

### 2.7. Homology Modeling and Molecular Dynamics Simulation

A swiss-model online database was used for the homology modeling of each target protein. Subsequently, the predicted models were validated using the Ramachandran plot [66]. The COFACTOR [67] server was employed for the prediction of the binding site in the generated models. Moreover, the model was checked for stability by molecular dynamics (MD) simulation methodology using AMBER v2014 software package [68]. The LEaP module was used to add the missing polar/non-polar hydrogen atoms and counterions (Na^+^ and Cl^−^) were added to neutralize the overall system. Next, a solvated octahedral box of transferable intermolecular potential with 3 points (TIP3P) water model (10.0 Å buffer) was used to sandwich the system in a water environment. Bonds involving hydrogen atoms were constrained with the SHAKE algorithm [69]. All MD simulations were done by the CUDA version of PMEMD in GPU cores of NVIDIA^®^ Tesla K80 [68]. The NPT ensemble at 298 K, 1 bar, and an integration time step of 2 fs was used to integrate the equations of motion. An Anderson-like temperature coupling scheme was used to control the temperature and imaginary “collisions” were randomized by the velocities at a distribution corresponding to simulation temperature every 1000 steps. Pressure control was performed using Berendsen barostat with the pressure relaxation time set to 1.0 ps. A cut-off 8.0 Å was used for Lennard–Jones interactions and the short-range electrostatic interactions.

## 3. Results and Discussion

### 3.1. Identification of Essential and Non-Host Homologous Proteins in Pathogens

**Figure 1:**
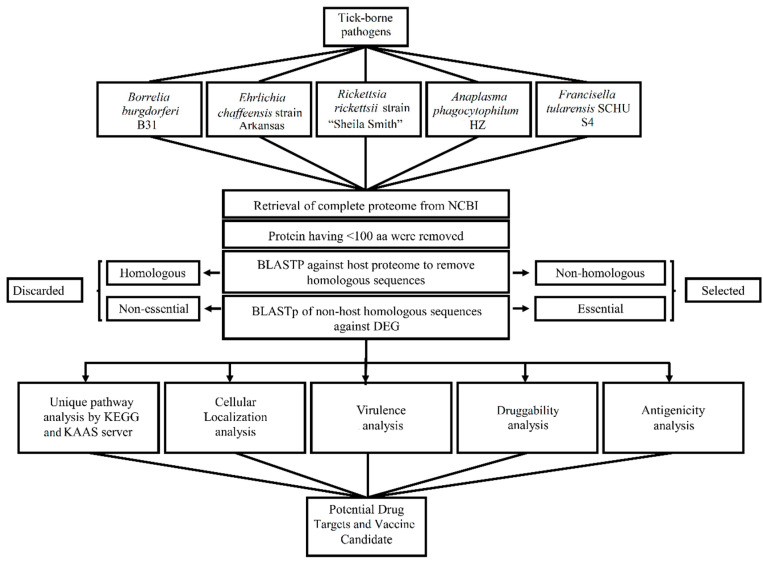
Outline of subtractive proteome analysis of Tick-borne pathogens.

To our knowledge, the subtractive analysis performed in this study is the first computational report to characterize and identify novel therapeutic targets for the control of TBPs. To predict unique proteins as drug targets and vaccine candidates within the proteome of a pathogen, subtractive proteomics has been reported among the most powerful approaches for unique yet uncharacterized sequences as possible therapeutic targets [18,25,33,70,71,72,73,74,75]. The objective of the current study was to predict novel drug targets and vaccine candidates based on subtractive proteomics approach against *B. burgdorferi* B31, *E. chaffeensis* str. Arkansas, *R. rickettsii* str. “Sheila Smith”, *A. phagocytophilum* HZ, and *F. tularensis* SCHU S4. The entire proteomes of selected TBPs were scanned to obtain a group of essential and non-host homologous proteins. Among them, cytoplasmic proteins were predicted as putative drug targets and a membrane-bound protein as a vaccine candidate. This membrane-bound protein may be a capable vaccine candidate for controlling infections caused by TBPs. The entire model of this subtractive analysis is given in the flow chart below (Figure 1).

Complete proteomes of selected pathogens, including *B. burgdorferi* B31 (1391 proteins), *E. chaffeensis* str. Arkansas (889 proteins), *R. rickettsii* str. “Sheila Smith” (1246 proteins), *A. phagocytophilum* HZ (1048 proteins), and *F. tularensis* SCHU S4 (1556 proteins), were retrieved and subjected to the CD-HIT algorithm to remove paralogous sequences [61]. A 40% similarity was chosen as a cut-off to maintain a very stringent selection criteria for the identification of the most effective targets. It has been widely accepted to set a 40% sequence identity as a cut-off to maintain a rigid criterion to remove duplicate proteins [31,45,71,76,77]. This is because protein sequence databases are incredibly redundant, and this redundancy occurs when several similar data are deposited from different regions [78]. The inclusion of similar sequences in individual-specific analyses mostly introduces undesirable biases [38,39]. Duplicate proteins and proteins with less than 100 amino acids were also excluded, and this has been previously documented [18,79,80]. A set of non-paralogous proteins was generated for further analysis based on the assumption that these proteins may be essential for pathogen survival [80,81]. The identified non-paralogous proteins were 1181 out of 1391 in *B. burgdorferi* B31, 846 out of 889 in *E. chaffeensis* str. Arkansas, 830 out of 1246 in *R. rickettsii* str. “Sheila Smith”, 712 out of 1048 in *A. phagocytophilum* HZ, and 1295 out of 1556 in *F. tularensis* SCHU S4. The non-redundant data set was further filtered, and only those proteins which had a sequence similarity less than 30% or no significant similarity with the host (*H. sapiens* and *B. taurus*) proteome were targeted. Further, an NCBI BLASTp search with a threshold expectation value of (E-value) 10^−5^ with the host (*H. sapiens* and *B. taurus*) was used, and sequences that showed no similarity with the host were selected. The resultant data set revealed non-host homologous proteins of pathogens. Non-host homologous proteins were 765 in *B. burgdorferi* B31, 793 in *E. chaffeensis* str. Arkansas, 409 in *R. rickettsii* str. “Sheila Smith”, 105 in *A*. *phagocytophilum* HZ, and 185 in *F. tularensis* SCHU S4.

Essential proteins are regularly required to support the basic cellular functions of micro-organisms and are essential for the survival of a pathogen [76,82]. A potent drug target must be an essential protein possessing features required for the survival and existence of a pathogen [75]. A BLASTp search for the non-homologous proteins of selected pathogens against the DEG database was done to screen out the essential proteins [43,83]. The queried proteins having a homologous hit in DEG was 34 in *B. burgdorferi* B31, 113 in *E. chaffeensis* str. Arkansas, 76 in *R. rickettsii* str. “Sheila Smith”, 105 in *A. phagocytophilum* HZ, and 185 in *F. tularensis* SCHU S4. All these predicted sets of essential proteins were found to be involved in metabolic pathways (Table 1).

### 3.2. Pathogens Unique Metabolic Pathways and Subcellular Localization

The predicted novel metabolic pathways in all TBPs were 66, and among them, 14 were in *R. rickettsii* str. “Sheila Smith”, 13 in *B. burgdorferi* B31, 8 in *E. chaffeensis* str. Arkansas, 6 in *A. phagocytophilum* HZ, and 25 in *F. tularensis* SCHU S4. A total of 61 proteins were found to be involved in metabolic pathways that are unique to TBPs and having no similarity with the host (*H. sapiens* and *B. taurus*) proteome. The unique metabolic pathways included the quorum-sensing metabolic pathway, two-component system, lysine biosynthesis, flagellar assembly, bacterial secretion system, monobactam biosynthesis, and the peptidoglycan biosynthesis (Table 2). These unique metabolic pathways contain essential proteins necessary for the survival, virulence, and pathogenicity of TBPs that can be used as drug targets and vaccine candidates.

**Figure 2:**
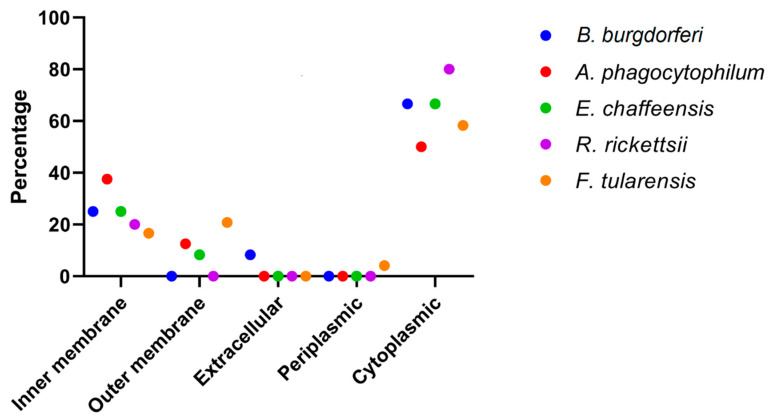
Subcellular localization of target proteins showing the majority are cytoplasmic. Borrelia burgdorferi (*B. burgdorferi*), Anplasma phagocytophilum (*A. phagocytophilum*), Ehrlichia chaffeensis (*E. chaffeensis*), Rickettsia rickettsii (*R. rickettsii*), Francisella tularensis (*F. tularensis*).

For the prediction of effective and suitable drug targets and vaccine candidates, it is vital to find the protein’s subcellular localization [84]. Extracellular, membrane-bound, and cytoplasmic proteins can be used as vaccine candidates and drug targets, respectively [18,51]. In *B. burgdorferi* B31, the CELLO server analysis predicted 8/12 (66.66%) proteins were cytoplasmic, 3/12 (25%) inner membrane, and 1/12 (8.33%) extracellular. In *A. phagocytophilum* HZ, 4/8 (50%) were cytoplasmic, 3/8 (37.5%) inner membrane, and 1/8 (12.55%) outer membranes. In *E. chaffeensis* str. Arkansas, 8/12 (66.6%) were cytoplasmic, 1/12 (8.33%) outer membrane, and 3/12 (25%) inner membrane. In *R. rickettsii* str. “Sheila Smith”, 4/5 (80%) were cytoplasmic, and 1/5 (20%) inner membrane. In *F. tularensis* SCHU S4, 14/24 (58.33%) were cytoplasmic, 5/24 (20.83%) outer membrane, 4/24 (16.66%) inner membrane, and 1/24 (4.16%) periplasmic (Figure 2). Cytoplasmic proteins have been suggested as favorable drug targets compared to membrane-bound proteins, because the latter often faces problems during purification [85].

### 3.3. Functional Analysis of Unique Pathways

Comparative analysis of the metabolic pathways of TBPs against the host (*H. sapiens* and *B. taurus*) revealed 66 unique pathways in TBPs having no similarities with the host. The KO list of TBPs proteins provided by the KAAS server was searched against each pathogen pathway to screen the unique essential proteins involved in unique pathways. Among them, 12 unique pathways—such as quorum-sensing, two-component system, and lysine biosynthesis in *A. phagocytophilum* HZ; flagellar assembly in *B. burgdorferi* B31; bacterial secretion system and monobactam biosynthesis in *E. chaffeensis* str. Arkansas; quorum-sensing and peptidoglycan biosynthesis in *F. tularensis* SCHU S4; and the two-component system in *R. rickettsii* str. “Sheila Smith”—have unique essential proteins having no similarities with host pathways (Table 2).

Proteins present in the quorum-sensing pathway are responsible for the bioluminescence, sporulation, competence, antibiotic production, biofilm formation, and virulence factors secretion [86,87,88]. Two of the target proteins, preprotein translocase subunit SecY and preprotein translocase subunit SecG protein, are present in the quorum-sensing pathway of *A. phagocytophilum* HZ and *F. tularensis* SCHU S4, respectively, which can be used as potential drug targets. The two-component system pathway, essential for the growth and survival in adverse environmental conditions, is ubiquitous in bacteria and has been reported to be involved in virulence [89,90]. The chromosomal replication initiator protein DnaA (dnaA) and cytochrome d ubiquinol oxidase subunit 1 protein are present in the two-component system pathway of the *A. phagocytophilum* HZ and *R. rickettsii* str. “Sheila Smith”, respectively. The peptide cross-linking in the peptidoglycan layer of bacteria plays a central role in pathogenesis. Inhibitors of peptidoglycans form a significant class of antibiotics and have been demonstrated as probable drug targets [91,92]. The biosynthesis of peptidoglycan involves various ADP forming ligases, such as MurA, MurC, MurD, MurE, and MurF, which catalyze the successive additions of l-alanine, d-glutamate, a diamino acid, and d-alanine-d-alanine to UDP-*N*-acetylmuramic acid [93]. Both UDP-*N*-acetylmuramate-l-alanine ligase (murC) and phospho-*N*-acetylmuramoyl-pentapeptide-transferase (murE) are present in the peptidoglycan pathway of the *F. tularensis* SCHU S4. These drug targets, which inhibit peptidoglycan biosynthesis, have the potential to control pathogens and minimize microbe-generated pathogenicity [77]. The general secretion (Sec) and twin-arginine translocation (Tat) pathways are the bacterial secretion system, most used to transport proteins across the cytoplasmic membrane [94]. Pathogens require a functional Tat pathway for virulence during infection, survival, and other physiological functions [95,96,97]. Similarly, the twin-arginine translocase subunit TatC present in the bacterial secretion system pathway, and aspartate kinase in the monobactam biosynthesis pathway of *E. chaffeensis* str. Arkansas is required for survival and virulence. Aspartate-semialdehyde dehydrogenase is present in the lysine biosynthesis pathway of *A. phagocytophilum* HZ. Several proteins of the flagellar assembly pathway are involved in protein export, especially in the export of VFs [98]. The proteins UDP-*N*-acetylmuramoyl-tripeptide-d-alanyl-d-alanine ligase and Flagellar secretion chaperone FliS are present in the flagellar assembly pathway of *B. burgdorferi* B31.

All the predicted 12 target proteins present in the unique pathways of TBPs have no similarities with the host pathways (*H. sapiens* and *B. taurus*). Thus, proteins involved in these pathways are potential drug targets, and their inhibition will increase the susceptibility of TBPs to various drugs (Table 2).

### 3.4. Druggability and Virulence Analysis for the Identification of Potential Drug Targets and Vaccine Candidates

To evaluate the druggability potential, the shortlisted essential proteins were subject to BLASTp against the FDA approved drugs. A total of fifteen proteins from all pathogens were predicted to be druggable. For instance, there were four protein targets (i.e., chitibiose transporter protein ChbA, FLiS, flagellar hook capping protein, UDP-*N*-acetylenolpyruvoyl glucosamine reductase) in *B. burgdorferi* B31, three-drug targets (i.e., twin-arginine translocase subunit TatC, preprotein translocase subunit SecA, and aspartate kinase) in *E. chaffeensis* str. Arkansas, one drug target (i.e., cytochrome d ubiquinol oxidase subunit I) in *R. rickettsii* str. “Sheila Smith”, four drug targets (i.e., UDP-*N*-acetylmuramate-l-alanine ligase, preprotein translocase subunit SecG, preprotein translocase subunit SecY, and UDP-*N*-acetylmuramoylalanyl-d-glutamate-2,6-diaminopimelate ligase) in *F. tularensis* SCHU S4, and three-drug targets (i.e., preprotein translocase subunit SecY, chromosomal replication initiator protein DnaA, and aspartate-semialdehyde dehydrogenase) in *A. phagocytophilum* HZ. Screening of VFs has been a promising option for the prediction of therapeutic targets [99]. To find virulency, the fifteen predicted protein targets of all pathogens were subject to BLASTp against the core data set (R1) of the VFDB. All target proteins were virulent except twin-arginine translocase subunit (TatC) from *E. chaffeensis* str. Arkansas (Table 2). The VFs inherited properties required for bacteria to adhere, colonize, invade, and conquer the host defense system and, thus, are considered as potential drug targets and vaccine candidates [100].

### 3.5. Screening of Essential, Non-Homologous Target Proteins Versus Gut Metagenome and Secondary Structure Analysis

The beneficial microbes that reside in the human digestive tract constitute gut microbiota. There are trillions of microbes that reside symbiotically in a human intestine [101,102]. These microbes contribute to ferment undigested carbohydrates and produce energy, preventing harmful species growth, and enhance the functions of the immune system in the residing host [101]. To exclude those proteins found in human gut flora, TPBs proteins were subject to BLASTp against the human microbiome project database. After metagenomics, eleven protein targets were found to have no similarity with the gut metagenome of the host and were considered as final target proteins. The eleven target proteins included: twin-arginine translocase subunit TatC, aspartate kinase, UDP-*N*-acetylmuramate-l-alanine ligase, preprotein translocase subunit SecG, preprotein translocase subunit SecY, UDP-*N*-acetylmuramoylalanyl-d-glutamate-2,6-diaminopimelate ligase, preprotein translocase subunit SecY, chromosomal replication initiator protein DnaA, aspartate-semialdehyde dehydrogenase, UDP-*N*-acetylmuramoyl-tripeptide-d-alanyl-d-alanine ligase, and flagellar protein FLiS (Table 2). These essential, non-host homologous, and virulent target proteins can be used as potential drug targets and vaccine candidates.

The predicted secondary structure drawn using SOPMA revealed the percentage of the α-helix, extended strand, β-turn, and random coil in each target protein (Table 3). The confidence of prediction observed throughout the predicted secondary structures was high, and a high percentage of α-helices was found in most of the target proteins. For instance, the α-helices contents were found to be 59.31% in FLiS protein. Most of the transmembrane proteins, especially those present in the cytoplasmic membrane, are solely constituted by α-helices. The extended strands or beta-sheets linked to the α-helices may construct the external transmembrane regions, thus providing stability to these proteins [103,104,105,106].

### 3.6. Phylogenetic Analysis

**Figure 3:**
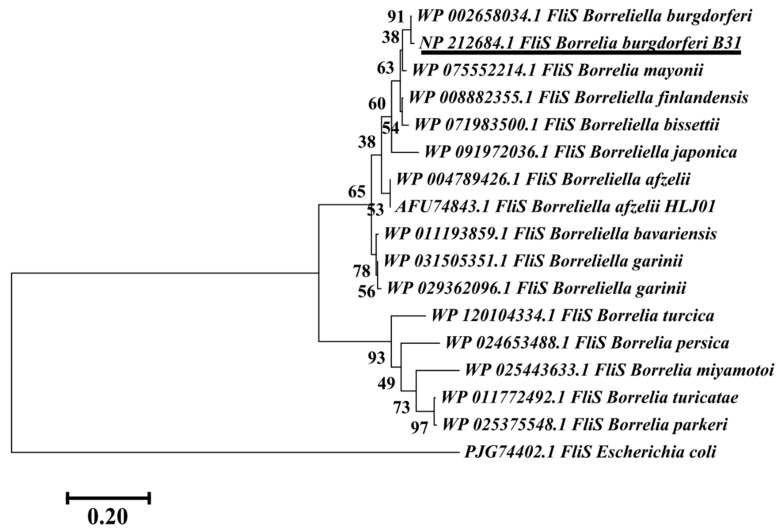
The neighbor-joining tree was constructed for the amino acid sequence of vaccine candidates of *B. burgdorferi* B31 protein. The target sequence of the vaccine candidate is underlined and support values (bootstrapping values) are indicated at each node. Species names are followed by accession number and the outgroup used to root the tree was *Escherichia coli* FliS sequence. The bar represents 0.20 substitutions per site.

The phylogenetic relationship is crucial for understanding the evolution and background history of various proteins. Nearly all proteins have structural similarities with other proteins and in some cases, share a common evolutionary origin. To determine the evolutionary relationship of the predicted vaccine candidate (FLiS protein), a neighbor-joining tree was constructed [65] which showed a 91% bootstrapping support value (Figure 3). All sequences were clustered together which suggested that this protein is highly conserved among various strains of *B. burgdorferi* and may play a functional role in pathogen survival, propagation, transmission, and pathogenesis. Further, the FLiS protein is present in TBP (*B. burgdorferi* B31) and other pathogens; it may serve as a universal vaccine by eliciting an immune response against several infectious agents [107,108].

### 3.7. Characterization of Drug Targets and Vaccine Candidates

**Figure 4:**
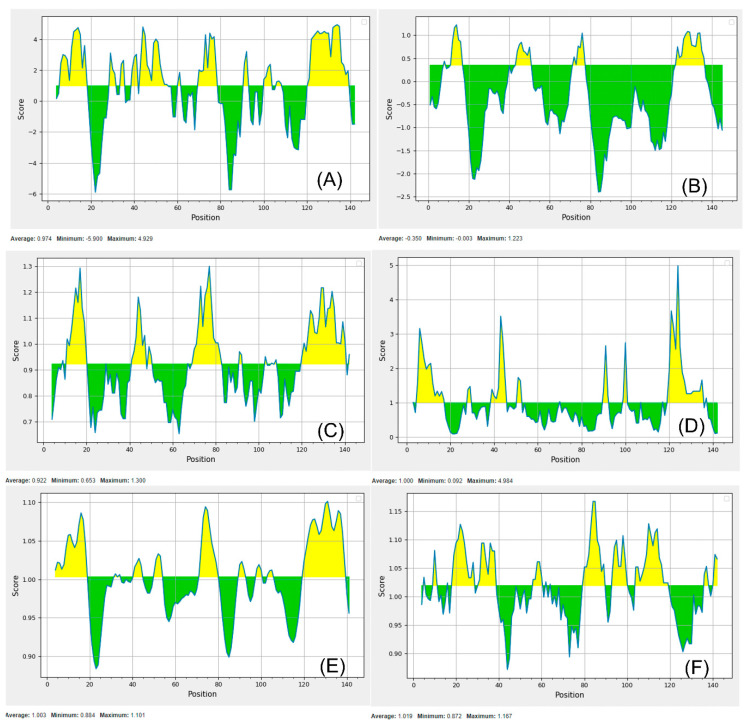
B-cell epitope in the FLiS protein based on Parker hydrophilicity prediction (**A**). Bepipred linear epitope (**B**). Chou and Fasman β-turn prediction (**C**). Emini surface accessibility prediction (**D**). Karplus and Schulz flexibility prediction (**E**). Kolaskar and Tongaonkar antigenicity (**F**). The x-axis and y-axis represent the sequence position and corresponding antigenic properties score, respectively. The threshold level was set as default parameter of the server. The regions shown in yellow color above the threshold value were predicted as B-cell epitope.

Among the twelve targets after cellular localization, five proteins were cytoplasmic, five inner membranes and only one protein was each outer membrane and extracellular. In the adhesion and invasion mechanism during the host–pathogen interaction, outer membrane proteins played a significant role in invading a host cell and entering the tissue [21]. Comparatively, it was evident from previous reports that outer membrane proteins are vaccine candidates and that cytoplasmic proteins are drug targets [21,109]. It is well known that exported proteins are the prominent molecules of interaction with cells infected by pathogens; therefore, they are potential candidates for vaccine targets [110,111,112,113,114]. The antigenicity and allergenicity analysis of target proteins revealed that eight among them were antigenic while the remaining four proteins were non-antigenic and all the target proteins were non-allergen (Table 2). The FLiS protein in *B. burgdorferi* B31 has several antigenic epitopes having the potential as a vaccine candidate. The extracellular protein (FLiS; UniProt ID: O51500_BORBU, accession no, NP_212684.1, KEGG ID: BBU02040) was found with a high antigenic score of 0.42 as well as human non-allergen. Potential T-cell epitopes were predicted within the FLiS protein for the prediction of an epitope-based subunit vaccine. The molecular weight of the FliS protein was 16.45 kDa, while the theoretical pI was measured as 9.20 which indicated that this protein should have a negative charge. The half-life of the vaccine was expected to be more than 10 h in *E. coli* in vivo. The estimated rate of extinction and an aliphatic index were 15,470 and 115.66, respectively. The protein’s computed GRAVY value was −0.253 while the index of instability (34.07) classified the protein as stable. The results of predicted B-cell epitopes of FLiS protein are shown in (Figure 4).

### 3.8. Homology Modeling and Molecular Dynamic Simulation

**Figure 5:**
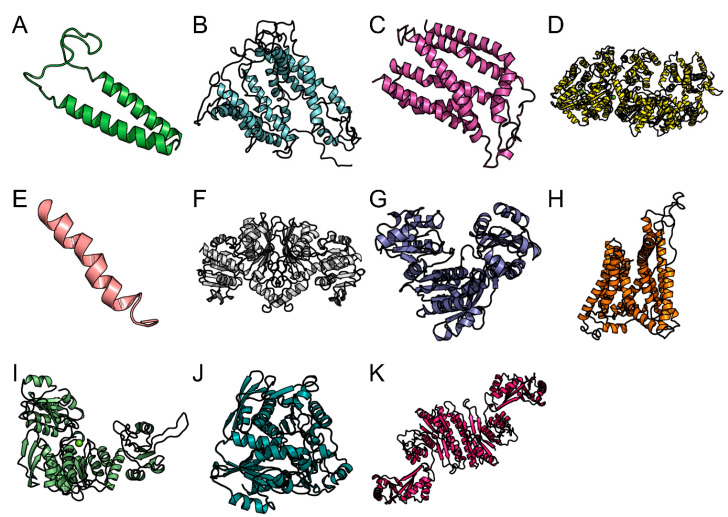
Predicted 3D structure of drug target proteins, preprotein translocase subunit SecG (*F. tularensis* SCHU S4) (**A**). Preprotein translocase subunit SecY (*F. tularensis* SCHU S4) (**B**). Twin-arginine translocase subunit TatC (*E. chaffeensis* str. Arkansas) (**C**). Chromosomal replication initiator protein DnaA (*A. phagocytophilum* HZ) (**D**). Cytochrome d ubiquinol oxidase subunit II (*R. rickettsii* str. “Sheila Smith”) (**E**). Aspartate-semialdehyde dehydrogenase (*A. phagocytophilum* HZ) (**F**). UDP-*N*-acetylmuramoylalanyl-d-glutamate-2,6-diaminopimelate ligase (*F. tularensis* SCHU S4) (**G**). Preprotein translocase subunit SecY (*A. phagocytophilum* HZ) (**H**). UDP-*N*-acetymuramoyl-tripeptide (*B. burgdoferri* B31) (**I)**. UPD-*N*-acetymuramate-l-alanine ligase (*F. tularensis* SCHU S4) (**J**). Aspartate kinase (*E. chaffeensis* str. Arkansas) (**K**).

**Figure 6:**
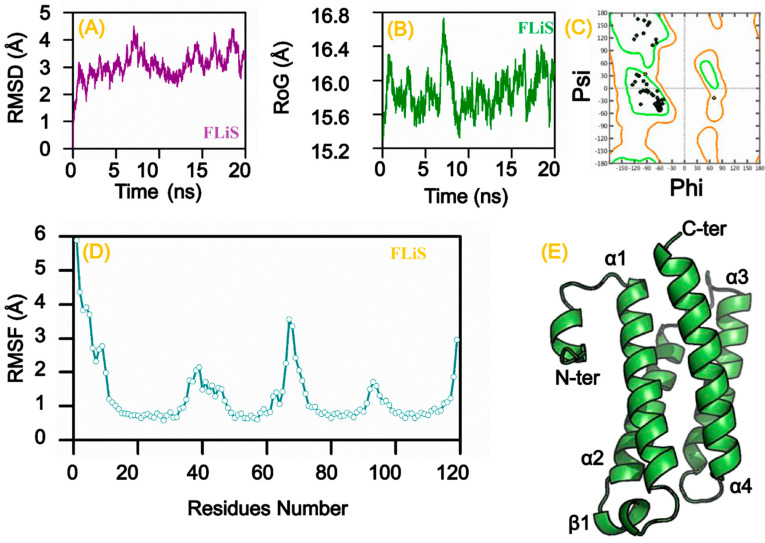
Molecular dynamics simulation for vaccine target (FLiS protein) (*B. burgdorferi* B31). Root Mean Square Deviation (RMSD) (**A**). Radius of Gyration (RoG) (**B**). Validation by Ramachandran plot (**C**). Root Mean Square Fluctuation (RMSF) (**D**). Tertiary structure prediction (**E**).

Structure similarity search for *B. burgdorferi* B31 protein UDP-n-acetylmuramoyl-tripeptide-D-alanyl-D-alanine ligase showed a 27% identity with crystal structure of unliganded CH59UA, the inferred unmutated ancestor of the RV144 anti-HIV antibody lineage producing CH59 Protein Data Bank (PDB ID 4QF5.1) (Figure 5I) and FLiS showed a 33.3% identity with a flagellar export chaperone in complex with its cognate binding partner from Aquifex aeolicus (PDB ID 1ORY.1) (Figure 6E). The *A. phagocytophilum* HZ protein preprotein translocase subunit SecY showed a 43.36% identity with crystal structure of the TEPC15-Vk45.1 anti-2-phenyl-5-oxazolone NQ16-113.8 scFv in complex with phOxGABA (PDB ID 3J45.1) (Figure 5H), chromosomal replication initiator protein DnaA showed 35.53% identity with AMPPCP-bound DnaA from *A. aeolicus* (PDB ID 2HCB.1) (Figure 5D), and aspartate-semialdehyde dehydrogenase showed 31.68% identity with aspartate semialdehyde dehydrogenase complexed with glycerol and sulfate from *Mycobacterium tuberculosis* H37Rv (PDB ID 3VOS) (Figure 5F). The *E. chaffeensis* str. Arkansas protein twin-arginine translocase subunit TatC showed 34.62% identity with twin arginine translocase receptor-Tatc In DDM from A. aeolicus (PDB ID 4HTT.1) (Figure 5C) and aspartate kinase showed 44.31% identity with aspartate kinase from *Synechocystis* species (PDB ID 3L76.1) (Figure 5K). The *R. rickettsii* str. “Sheila Smith” protein cytochrome d ubiquinol oxidase subunit II showed 12% identity with alternative complex iii from Rhodothermus marinus (PDB ID 6F0K.1) (Figure 5E). The *F. tularensis* SCHU S4 protein preprotein translocase subunit SecG showed a 52.11% identity with quaternary complex between SRP, SR, and SecYEG bound to the translating ribosome from *E. coli* (PDB ID 5NCO.1) (Figure 5A), UDP-*N*-acetylmuramate-l-alanine ligase showed a 37.27% identity with 2.25 angstrom resolution crystal structure of UDP-n-acetylmuramate--L-alanine ligase (murC) from Yersinia pestis CO92 in complex with AMP (PDB ID 4HV4.2) (Figure 5J), preprotein translocase subunit SecY showed a 62.32% identity with EM fitted model of bacterial holo-translocon from *E. coli* (PDB ID 5MG3.1) (Figure 5B), and UDP-*N*-acetylmuramoylalanyl-d-glutamate-2, 6-diaminopimelate ligase showed a 29.94% identity with *Staphylococcus aureus* MurE with UDP-MurNAc- Ala-Glu-Lys and ADP (PDB ID 4C12) (Figure 5G). A total of five models were generated for every drug target and vaccine candidate. However, molecular dynamics simulation was done only for the predicted vaccine candidate. The generated model of the vaccine candidate, FLiS protein (*B. burgdorferi* B31), was validated, and the evaluation of Psi and Phi dihedral angles for FLiS model revealed that high residues lie in favored regions as compared to allowed regions (Figure 6C). The details of the binding site and binding site residues of vaccine candidate (FliS protein) are shown in (Table 4).

With constant improvement in algorithm design for simulations, MD simulations have played an essential role in the development of novel therapeutics [115]. The FLiS structure was simulated in an explicit water environment for 20 ns. The deviation of the backbone atoms was examined by the root-mean-square deviation (RMSD). Consequently, the results of the backbone deviation relative to the original structures revealed that the simulation time of 20 ns is enough to reach the equilibration at temperature 298 K. It was observed from the RMSD graphs that the FLiS system initially behaves systematically steady (~3 Å) till 6 ns. However, this steady behavior dramatically increased and then oscillated around (~4 Å) until 20 ns (Figure 6A). To understand the effect of specific residues in the FLiS system, we analyzed the root-mean-square fluctuations (RMSF) (Figure 6D). The results revealed a high fluctuation in some residues (residues 38–45 and 65–78) which suggested that these residues might play a crucial role in flagellin recognition [116]. The compactness of the system was analyzed through radius of gyration (RoG) during MD simulation which showed high compactness during 7 ns, while showing local compactness afterward (Figure 6B). These results delineate that the FLiS protein possesses a highly dynamic N-terminal region, which is appended to the standard four-helix bundle structure, and further indicates that the FLiS could be used as a potential vaccine candidate against TBPs.

## 4. Conclusions and Future Directions

Subtractive proteomics is a rapid approach for the screening of drug targets and vaccine candidates against a pathogen provided both the pathogen and host proteomes are available. We applied a subtractive proteomics approach to find essential and non-host homologous protein targets in the proteome of TBPs which can be used as potential drug targets and vaccine candidates. Further analysis of shortlisted targets, such as different metabolic pathways proteins, subcellular localization of targets, antigenicity, allergenicity, and druggable properties, revealed eleven drug targets (cytoplasmic proteins) and one vaccine candidate (membrane-bound protein). Inhibiting proteins involved in these metabolic pathways will increase the susceptibility of TBPs to various drugs. The identified FliS protein has immunogenic and allergenic potential, and further studies on various aspects of this protein will help in understanding its diverse functions, development of a suitable vaccine against TBPs, and treatment of allergenic diseases caused by TBPs. This study will facilitate the development of drug targets and vaccine candidates against TBPs and may play a role in the prediction of targets against other pathogens. Furthermore, the proposed vaccine needs to be validated experimentally in an animal model by effective immunological methods to ensure the control of TBPs.

## Figures and Tables

**Table 1:** Subtractive proteome analysis and proteins involved in metabolic pathways of selected tick-borne pathogens.

Step Number	Steps	*Borrelia burgdorferi* B31	*Ehrlichia chaffeensis Arkansas*	*Rickettsia rickettsii* Strain “Sheila Smith”	*Anaplasma phagocytophilum HZ*	*Francisella tularensis* SCHU S4
		Host				
		*Homo sapiens*	*Homo sapiens*	*Bos taurus*	*Homo sapiens*	*Homo sapiens*
1.	Total proteome	1391	889	1246	1048	1556
2.	Duplicates (>60% identical) in CD-HIT ^a^	1181	846	830	712	1295
3.	Non-homologs	765	793	409	453	788
4.	Essential proteins in DEG ^b^	34	113	76	105	185
5.	Unique metabolic pathway KEGG ^c^	13	8	14	6	25
6.	Essential proteins involved KEGG and KAAS ^d^	12	12	5	8	24
7.	Druggability with cut-off E-value 10^−5^	4	3	1	3	4
8. *	Gut metagenomics with cut-off E-value 10^−5^	2	2	-	3	4

* Gut metagenomics were checked for the Homo sapiens host. ^a^ CD-HIT: Cluster Database at High Identity with Tolerance against nr database. ^b^ DEG: database of essential gene contains essential gene required for survival. ^c^ KEGG; Kyota Encyclopedia of genes and genomes contain organism pathways. ^d^ KAAS: KEGG Automatic Annotation Server provides functional annotation of genes.

**Table 2:** List of biological and molecular functions, non-host homologous, essential pathways, subcellular localization, druggability, and antigenicity of identified targets.

Name of Protein	NCBI Protein ID	Pathways	KO Number	KEGG ID	Biological Process	Molecular Function	Drug Bank ID	Drug Name	Chemical Formulae	Drug Group	Virulent	Localization	Antigenicity Score	Antigenicity	Allergenicity
Preprotein translocase subunit SecY *A. phagocytophilum*	WP_011450434.1	Quorum-sensing, *A. phagocytophilum* HZ	K03076	aph02024	Protein transport, translocation	None predicted evidence	DB06292 DB08907 DB09038	Dapagliflozin Canagliflozin Empagliflozin	C_21_H_25_ClO_6_ C_24_H_25_FO_5_S C_23_H_27_ClO_7_	Approved Approved Approved	Yes	Inner membrane	0.4083	Antigen	Non-allergen
Chromosomal replication initiator protein DnaA. *A. phagocytophilum*	WP_011450597.1	Two-component system, *A. phagocytophilum* HZ	K02313	aph02020	DNA replication	DNA-binding	DB00173	Adenine	C_5_H_5_N_5_	Approved, nutraceutical	Yes	Cytoplasmic	0.3982	Non-Antigen	Non-allergen
Aspartate-semialdehyde dehydrogenase *A. phagocytophilum*	WP_011451356.1	Lysine biosynthesis, *A. phagocytophilum* HZ	K00133	aph00300	Cellular amino acid biosynthetic process	Oxidoreductase activity	DB00181 DB00996 DB02530	Baclofen Gabapentin gamma-Aminobutyric acid	C_10_H_12_ClNO_2_ C_9_H_17_NO_2_ C_4_H_9_NO_2_	Approved Approved, investigational Approved, investigational	Yes	Outer membrane	0.3987	Non-Antigen	Non-allergen
1UDP-n-acetylmuramoyl-tripeptide--D-alanyl-D-alanine ligas *B. burgdorferi* B31	NP_212438.1	Flagellar assembly, *B. burgdorferi* B31	K01929	bbu02040	Peptidoglycan biosynthetic process	ATP binding	DB04272	Citric acid	C_6_H_8_O_7_	Approved, nutraceutical, Vet approved	Yes	Cytoplasmic	0.2503	Non-Antigen	Non-allergen
FliS *B. burgdorferi* B31	NP_212684.1	Flagellar assembly, *B. burgdorferi* B31	K02422	bbu02040	Bacterial-type flagellum assembly	None predicted evidence	DB00120	l-phenylalanine	C_9_H_11_NO_2_	Approved, Investigational, nutraceutical	Yes	Extracellular	0.4200	Antigen	Non-allergen
Twin-arginine translocase subunit TatC *Ehrlichia chaffeensis*	WP_011452677.1	Bacterial secretion system, *Ehrlichia chaffeensis* Arkansas	K03118	ech03070	Protein transport by the Tat complex	Protein transmembrane transporter activity	DB01277 DB13173	Mecasermin cerliponase alfa	C_331_H_518_N_94_O_101_S_7_ Not Available	Approved, investigational Approved, investigational	No	Inner membrane	0.8915	Antigen	Non-allergen
Aspartate kinase *E. chaffeensis*	WP_011452940.1	Monobactam biosynthesis, *E. chaffeensis* Arkansas	K00928	ech00261	Lysine biosynthetic process via diaminopimelate	Aspartate kinase activity	DB11638	Artenimol	C_15_H_24_O_5_	Experimental, investigational	Yes	Cytoplasmic	0.5049	Antigen	Non-allergen
Preprotein translocase subunit SecG *F. tularensis* SCHU S4	YP_169156.1	Quorum sensing, *F. tularensis*. S4	K03075	ftu02024	Protein secretion	Protein translocase activity	DB00887 DB01016 DB01050 DB04941 DB08820 DB09213 DB09280	Bumetanide Glyburide Ibuprofen Crofelemer Ivacaftor Dexibuprofen Lumacaftor	C_17_H_20_N_2_O_5_S C_23_H_28_ClN_3_O_5_S C_13_H_18_O_2_ Not Available C_24_H_28_N_2_O_3_ C_13_H_18_O_2_ C_24_H_18_F_2_N_2_O_5_	Approved Approved Approved Approved Approved, investigational Approved, investigational Approved	Yes	Inner membrane	0.7645	Antigen	Non-allergen
UDP-*N*-acetylmuramate-l-alanine ligase *F. tularensis* SCHU S4	YP_169292.1	Peptidoglycan biosynthesis, *F. tularensis* SCHU S4	K01924	ftu00550	Murein biosynthesis	Ligase activity	DB00157 DB09092	NADH Xanthinol	C_21_H_29_N_7_O_14_P_2_ C_13_H_21_N_5_O_4_	Approved, nutraceutical Approved, withdrawn	Yes	Cytoplasmic	0.4349	Antigen	Non-allergen
Preprotein translocase subunit SecY *F. tularensis* SCHU S4	YP_169394.1	Quorum sensing, *F. tularensis* SCHU S4	K03076	ftu02024	Protein transport	None predicted evidence	DB00313; DB05541	Valproic acid brivaracetam	C_8_H_16_O_2_ C_11_H_20_N_2_O_2_	Approved, investigational Approved, investigational	Yes	Cytoplasmic	0.6388	Antigen	Non-allergen
UDP-*N*-acetylmuramoylalanyl-d-glutamate-2,6-diaminopimelate ligase *F. tularensis* SCHU S4	YP_169464.1	Peptidoglycan biosynthesis, *F. tularensis* SCHU S4	K01928	ftu00550	Murein biosynthesis	Ligase activity	DB11638	Artenimol	C_15_H_24_O_5_	Experimental, investigational	Yes	Inner membrane	0.3851	Non-antigen	Non-allergen
Cytochrome d ubiquinol oxidase subunit II- *R. rickettsii* Sheila Smith	WP_012150506.1	Two-component system, *R. rickettsii* “Sheila Smith”	K00426	rri02020	Oxidation-reduction process	None predicted evidence	DB01221	Ketamine	C_13_H_16_ClNO	Approved, vet approved	Yes	Inner membrane	0.6850	Antigen	Non-allergen

**Table 3:** Secondary structure prediction of drug targets and vaccine candidates.

SOPMA ^a^	Flagellar Protein (FLiS, *B. burgdorferi*)	UDP-*N*-acetylmuramoyl-tripeptide-d-alanyl-d-alanine ligase *B. burgdorferi*	Preprotein Translocase Subunit SecY (*A. phagocytophilum*)	Chromosomal Replication Initiator Protein DnaA (*A. phagocytophilum*)	Aspartate-Semialdehyde Dehydrogenase (*A. phagocytophilum*)	Twin-Arginine Translocase Subunit TatC (*E. chaffeensis*)	Aspartate Kinase (*E. chaffeensis*)	Cytochrome d Ubiquinol Oxidase Subunit II (*R. rickettsii*)	Preprotein Translocase Subunit SecG (*F. tularensis*)	Preprotein Translocase Subunit SecY (*F. tularensis*)	UDP-*N*-acetylmuramate-l-alanine ligase (*F. tularensis*)	UDP-*N*-acetylmuramoylalanyl-d-glutamate-2,6-diaminopimelate ligase (*F. tularensis*)
α-helix	59.31%	38.36%	47.24%	54.03%	36.80%	55.42%	37.65%	50.74%	33.33%	47.62%	37.69%	42.17%
310 helices	0.00%	0.00%	0.00%	0.00%	0.00%	0.00%	0.00%	0.00%	0.00%	0.00%	0.00%	0.00%
Pi helix	0.00%	0.00%	0.00%	0.00%	0.00%	0.00%	0.00%	0.00%	0.00%	0.00%	0.00%	0.00%
Beta bridge	0.00%	0.00%	0.00%	0.00%	0.00%	0.00%	0.00%	0.00%	0.00%	0.00%	0.00%	0.00%
Extended strand	13.10%	22.84%	18.66%	12.64%	22.26%	15.66%	22.54%	15.34%	16.24%	17.91%	21.95%	18.79%
β-turn	3.45%	7.11%	8.06%	2.83%	6.53%	4.42%	7.19%	4.72%	2.56%	8.62%	7.98%	5.22%
Bend region	0.00%	0.00%	0.00%	0.00%	0.00%	0.00%	0.00%	0.00%	0.00%	0.00%	0.00%	0.00%
Random coil	24.14%	31.68%	26.04%	30.50%	34.42%	24.50%	32.61%	29.20%	47.86%	25.85%	32.37%	33.82%
Ambiguous states	0.00%	0.00%	0.00%	0.00%	0.00%	0.00%	0.00%	0.00%	0.00%	0.00%	0.00%	0.00%
Other states	0.00%	0.00%	0.00%	0.00%	0.00%	0.00%	0.00%	0.00%	0.00%	0.00%	0.00%	0.00%

^a^ SOPMA: Self Optimized Prediction Method from Multiple Alignment is a server used for secondary structure prediction.

**Table 4:** The predicted binding site for vaccine candidates and their interacting residues using the COFACTOR server.

Protein Name	Cscore ^LB^	PDB Hit	TM-Score	RMSD ^a^	IDEN ^a^	Cov.	BS-Score	Lig. Name	Predicted Binding Site Residues
NP_2126841	0.30	3asoC	0.697	2.53	0.034	0.891	0.94	CDL	24, 35, 112, 116
	0.10	3ag4C	0.696	2.54	0.034	0.891	1.01	CDL	20, 24, 28, 31, 119
	0.08	1ory0	0.952	0.95	0.336	0.975	1.47	III	8, 10, 13, 14, 19, 22, 24, 25, 28, 57, 60, 61, 64, 65, 68, 69, 70, 73, 74, 76, 77, 80, 83, 84, 85, 87, 106, 107, 109, 110, 113, 114, 117, 118, 120, 121
	0.02	2eikC	0.697	2.53	0.034	0.891	0.84	CD	26, 30, 108, 112

Cscore ^LB^: Confidence score (Cscore) for the ligand binding (LB). PDB: Protein Data Bank. TM-score: Two Model comparison score. RMSD ^a^: Root Mean Square Deviation is the RMSD between residue structurally aligned (a) by TM-align. IDEN ^a^: Identity in the structurally aligned (a) region. Cov: Represent the coverage. BS-score: Based on structural similarity. Lig Name: Ligand name. CDL: Cardiolipin.

## References

[1] Merino O., Alberdi P., Pérez de la Lastra J.M., de la Fuente J. (2013). Tick vaccines and the control of tick-borne pathogens. Front. Cell. Infect. Microbiol..

[2] Ogden N. (2013). Changing geographic ranges of ticks and tick-borne pathogens: Drivers, mechanisms and consequences for pathogen diversity. Front. Cell. Infect. Microbiol..

[3] Gondard M., Cabezas-Cruz A., Charles R.A., Vayssier-Taussat M., Albina E., Moutailler S. (2017). Ticks and tick-borne pathogens of the Caribbean: Current understanding and future directions for more comprehensive surveillance. Front. Cell. Infect. Microbiol..

[4] Ali A., Mulenga A., Da Silva Vaz I. (2020). Tick and Tick-Borne Pathogens: Molecular and Immune Targets for Control Strategies. Front. Physiol..

[5] Labruna M.B., Jorge R.S., Sana D.A., Jácomo A.T.A., Kashivakura C.K., Furtado M.M., Ferro C., Perez S.A., Silveira L., Santos T.S. (2005). Ticks (Acari: Ixodida) on wild carnivores in Brazil. Exp. Appl. Acarol..

[6] de la Fuente J., Estrada-Pena A., Venzal J.M., Kocan K.M., Sonenshine D.E. (2008). Overview: Ticks as vectors of pathogens that cause disease in humans and animals. Front. Biosci..

[7] Socolovschi C., Mediannikov O., Raoult D., Parola P. (2009). The relationship between spotted fever group *Rickettsiae* and Ixodid ticks. Vet. Res..

[8] Estrada-Peña A., Ayllón N., De La Fuente J. (2012). Impact of climate trends on tick-borne pathogen transmission. Front. Physiol..

[9] Pujalte G.G., Chua J.V. (2013). Tick-borne infections in the United States. Prim. Care.

[10] Bente D.A., Forrester N.L., Watts D.M., McAuley A.J., Whitehouse C.A., Bray M. (2013). Crimean-Congo hemorrhagic fever: History, epidemiology, pathogenesis, clinical syndrome and genetic diversity. Antivir. Res..

[11] Chmelař J., Kotál J., Kopeckỳ J., Pedra J.H., Kotsyfakis M. (2016). All for one and one for all on the tick–host battlefield. Trends Parasitol..

[12] Estrada-Peña A. (2008). Climate, niche, ticks, and models: What they are and how we should interpret them. Parasitol. Res..

[13] Dantas-Torres F. (2015). Climate change, biodiversity, ticks and tick-borne diseases: The butterfly effect. Int. J. Parasitol. Parasites Wildl..

[14] Hildebrandt A., Fritzsch J., Franke J., Sachse S., Dorn W., Straube E. (2011). Co-circulation of emerging tick-borne pathogens in Middle Germany. Vector Borne Zoonot. Dis..

[15] Parola P., Paddock C.D., Socolovschi C., Labruna M.B., Mediannikov O., Kernif T., Abdad M.Y., Stenos J., Bitam I., Fournier P.-E. (2013). Update on tick-borne rickettsioses around the world: A geographic approach. Clin. Microbiol. Rev..

[16] Otranto D., Dantas-Torres F., Giannelli A., Latrofa M.S., Cascio A., Cazzin S., Ravagnan S., Montarsi F., Zanzani S.A., Manfredi M.T. (2014). Ticks infesting humans in Italy and associated pathogens. Parasites Vectors.

[17] Brites-Neto J., Duarte K.M.R., Martins T.F. (2015). Tick-borne infections in human and animal population worldwide. Vet. World.

[18] Barh D., Tiwari S., Jain N., Ali A., Santos A.R., Misra A.N., Azevedo V., Kumar A. (2011). In silico subtractive genomics for target identification in human bacterial pathogens. Drug Dev. Res..

[19] Ali A., Tirloni L., Isezaki M., Seixas A., Konnai S., Ohashi K., da Junior I.S.V., Termignoni C. (2014). Reprolysin metalloproteases from *Ixodes persulcatus*, *Rhipicephalus sanguineus* and *Rhipicephalus microplus* ticks. Exp. Appl. Acarol..

[20] Hosen M.I., Tanmoy A.M., Mahbuba D.-A., Salma U., Nazim M., Islam M.T., Akhteruzzaman S. (2014). Application of a subtractive genomics approach for in silico identification and characterization of novel drug targets in *Mycobacterium tuberculosis* F11. Interdiscip. Sci..

[21] Solanki V., Tiwari V. (2018). Subtractive proteomics to identify novel drug targets and reverse vaccinology for the development of chimeric vaccine against *Acinetobacter baumannii*. Sci. Rep..

[22] Lee N.-H., Lee J.-A., Park S.-Y., Song C.-S., Choi I.-S., Lee J.-B. (2012). A review of vaccine development and research for industry animals in Korea. Clin. Exp. Vaccine Res..

[23] Sakharkar K.R., Sakharkar M.K., Chow V.T. (2004). A novel genomics approach for the identification of drug targets in pathogens, with special reference to *Pseudomonas aeruginosa*. In Silico Biol..

[24] Singh S., Malik B.K., Sharma D.K. (2007). Metabolic pathway analysis of *S. pneumoniae*: An in silico approach towards drug-design. J. Bioinform. Comput. Biol..

[25] Anishetty S., Pulimi M., Pennathur G. (2005). Potential drug targets in *Mycobacterium tuberculosis* through metabolic pathway analysis. Comput. Biol. Chem..

[26] Asif S.M., Asad A., Faizan A., Anjali M.S., Arvind A., Neelesh K., Hirdesh K., Sanjay K. (2009). Dataset of potential targets for *Mycobacterium tuberculosis* H37Rv through comparative genome analysis. Bioinformation.

[27] de la Fuente J., Contreras M. (2015). Tick vaccines: Current status and future directions. Expert. Rev. Vaccines.

[28] de la Fuente J., Kopáček P., Lew-Tabor A., Maritz-Olivier C. (2016). Strategies for new and improved vaccines against ticks and tick-borne diseases. Parasite Immunol..

[29] Allsop A.E. (1998). Bacterial genome sequencing and drug discovery. Curr. Opin. Biotechnol..

[30] Stumm G., Russ A., Nehls M. (2002). Deductive Genomics: A Functional Approach to Identify Innovative Drug Targets in the Post-Genome Era. Am. J. Pharm..

[31] Barh D., Kumar A. (2009). In silico identification of candidate drug and vaccine targets from various pathways in *Neisseria gonorrhoeae*. In Silico Biol..

[32] Ali A., Fernando Parizi L., Garcia Guizzo M., Tirloni L., Seixas A., da Silva Vaz I., Termignoni C. (2015). Immunoprotective potential of a *Rhipicephalus* (*Boophilus*) *microplus* metalloprotease. Vet. Parasitol..

[33] Amineni U., Pradhan D., Marisetty H. (2010). In silico identification of common putative drug targets in *Leptospira interrogans*. J. Chem. Biol..

[34] Kumar Jaiswal A., Tiwari S., Jamal S.B., Barh D., Azevedo V., Soares S.C. (2017). An in silico identification of common putative vaccine candidates against *Treponema pallidum*: A reverse vaccinology and subtractive genomics based approach. Int. J. Mol. Sci..

[35] Chan J.N., Nislow C., Emili A. (2010). Recent advances and method development for drug target identification. Trends Pharmacol. Sci..

[36] Madagi S., Patil V.M., Sadegh S., Singh A.K., Garwal B., Banerjee A., Talambedu U., Bhattacharjee B. (2011). Identification of membrane associated drug targets in *Borrelia burgdorferi* ZS7-subtractive genomics approach. Bioinformation.

[37] Maurya P.K., Singh S., Mani A. (2018). Comparative genomic analysis of *Rickettsia rickettsii* for identification of drug and vaccine targets: TolC as a proposed candidate for case study. Acta Trop..

[38] Cornish-Bowden A. (1983). The amino acid compositions of proteins are correlated with their molecular sizes. Biochem. J..

[39] Sikic K., Carugo O. (2010). Protein sequence redundancy reduction: Comparison of various method. Bioinformation.

[40] Li W., Godzik A. (2006). Cd-hit: A fast program for clustering and comparing large sets of protein or nucleotide sequences. Bioinformatics.

[41] Huang Y., Niu B., Gao Y., Fu L., Li W. (2010). CD-HIT Suite: A web server for clustering and comparing biological sequences. Bioinformatics.

[42] Altschul S.F., Gish W., Miller W., Myers E.W., Lipman D.J. (1990). Basic local alignment search tool. J. Mol. Biol..

[43] Zhang R., Lin Y. (2009). DEG 5.0, a database of essential genes in both prokaryotes and eukaryotes. Nucleic Acids Res..

[44] Sharma A., Pan A. (2012). Identification of potential drug targets in *Yersinia pestis* using metabolic pathway analysis: MurE ligase as a case study. Eur. J. Med. Chem..

[45] Mondal S.I., Ferdous S., Jewel N.A., Akter A., Mahmud Z., Islam M.M., Afrin T., Karim N. (2015). Identification of potential drug targets by subtractive genome analysis of *Escherichia coli* O157: H7: An in silico approach. Adv. Appl. Bioinform. Chem. AABC.

[46] Birhanu B.T., Lee S.-J., Park N.-H., Song J.-B., Park S.-C. (2018). In silico analysis of putative drug and vaccine targets of the metabolic pathways of *Actinobacillus pleuropneumoniae* using a subtractive/comparative genomics approach. J. Vet. Sci..

[47] Kanehisa M., Goto S. (2000). KEGG: Kyoto encyclopedia of genes and genomes. Nucleic Acids Res..

[48] Moriya Y., Itoh M., Okuda S., Yoshizawa A.C., Kanehisa M. (2007). The metabolic pathways were examined through KAAS (KEGG automatic annotation server): An automatic genome annotation and pathway reconstruction server. Nucleic Acids Res..

[49] Kanehisa M., Goto S., Sato Y., Kawashima M., Furumichi M., Tanabe M. (2014). Data, information, knowledge and principle: Back to metabolism in KEGG. Nucleic Acids Res..

[50] Kanehisa M., Sato Y., Kawashima M., Furumichi M., Tanabe M. (2016). KEGG as a reference resource for gene and protein annotation. Nucleic Acids Res..

[51] Yu C.-S., Chen Y.-C., Lu C.-H., Hwang J.-K. (2006). Prediction of protein subcellular localization. Proteins.

[52] Knox C., Law V., Jewison T., Liu P., Ly S., Frolkis A., Pon A., Banco K., Mak C., Neveu V. (2010). DrugBank 3.0: A comprehensive resource for ‘omics’ research on drugs. Nucleic Acids Res..

[53] Chen L., Yang J., Yu J., Yao Z., Sun L., Shen Y., Jin Q. (2005). VFDB: A reference database for bacterial virulence factors. Nucleic Acids Res..

[54] Larsen J.E.P., Lund O., Nielsen M. (2006). Improved method for predicting linear B-cell epitopes. Immunome Res..

[55] Kolaskar A.S., Tongaonkar P.C. (1990). A semi-empirical method for prediction of antigenic determinants on protein antigens. FEBS Lett..

[56] Emini E.A., Hughes J.V., Perlow D., Boger J. (1985). Induction of hepatitis A virus-neutralizing antibody by a virus-specific synthetic peptide. J. Virol..

[57] Karplus P.A., Schulz G.E. (1985). Prediction of chain flexibility in proteins. Naturwissenschaften.

[58] Chou P.Y., Fasman G.D. (1978). Empirical Predictions of Protein Conformation. Annu. Rev. Biochem..

[59] Rini J.M., Schulze-Gahmen U., Wilson I.A. (1992). Structural evidence for induced fit as a mechanism for antibody-antigen recognition. Science.

[60] Gasteiger E., Gattiker A., Hoogland C., Ivanyi I., Appel R.D., Bairoch A. (2003). ExPASy: The proteomics server for in-depth protein knowledge and analysis. Nucleic Acids Res..

[61] Wu G.D., Bushmanc F.D., Lewis J.D. (2013). Diet, the human gut microbiota, and IBD. Anaerobe.

[62] Geourjon C., Deleage G. (1995). SOPMA: Significant improvements in protein secondary structure prediction by consensus prediction from multiple alignments. Bioinformatics.

[63] Hall T.A. (1999). BioEdit: A user-friendly biological sequence alignment editor and analysis program for Windows 95/98/NT. Proceedings of the Nucleic Acids Symposium Series.

[64] Kumar S., Stecher G., Li M., Knyaz C., Tamura K. (2018). MEGA X: Molecular evolutionary genetics analysis across computing platforms. Mol. Biol. Evol..

[65] Tamura K., Stecher G., Peterson D., Filipski A., Kumar S. (2013). MEGA6: Molecular Evolutionary Genetics Analysis Version 6.0. Mol. Biol. Evol..

[66] Laskowski R.A., MacArthur M.W., Moss D.S., Thornton J.M. (1993). PROCHECK: A program to check the stereochemical quality of protein structures. J. Appl. Crystallogr..

[67] Roy A., Yang J., Zhang Y. (2012). COFACTOR: An accurate comparative algorithm for structure-based protein function annotation. Nucleic Acids Res..

[68] Salomon-Ferrer R., Götz A.W., Poole D., Le Grand S., Walker R.C. (2013). Routine microsecond molecular dynamics simulations with AMBER on GPUs. 2. Explicit solvent particle mesh Ewald. J. Chem..

[69] Ryckaert J.-P., Ciccotti G., Berendsen H.J.C. (1977). Numerical integration of the cartesian equations of motion of a system with constraints: Molecular dynamics of n-alkanes. J. Comput. Phys..

[70] Sharma V., Gupta P., Dixit A. (2008). In silico identification of putative drug targets from different metabolic pathways of *Aeromonas hydrophila*. In Silico Biol..

[71] Dutta A., Singh S.K., Ghosh P., Mukherjee R., Mitter S., Bandyopadhyay D. (2006). In silico identification of potential therapeutic targets in the human pathogen *Helicobacter pylori*. In Silico Biol..

[72] Georrge J.J., Umrania V. (2011). In silico identification of putative drug targets in *Klebsiella pneumonia* MGH78578. Indian J. Biotechnol..

[73] H Reddy P., P Reddy T. (2011). Mitochondria as a therapeutic target for aging and neurodegenerative diseases. Curr. Alzheimer Res..

[74] Shoukat K., Rasheed N., Sajid M. (2012). Subtractive genome analysis for in silico identification and characterization of novel drug targets in *C. trachomatis* STRAIN D/UW-3/Cx. Int. J. Curr. Res..

[75] Sarkar M., Maganti L., Ghoshal N., Dutta C. (2012). In silico quest for putative drug targets in *Helicobacter pylori* HPAG1: Molecular modeling of candidate enzymes from lipopolysaccharide biosynthesis pathway. J. Mol. Model..

[76] Rahman H., King R.M., Shewell L.K., Semchenko E.A., Hartley-Tassell L.E., Wilson J.C., Day C.J., Korolik V. (2014). Characterisation of a multi-ligand binding chemoreceptor CcmL (Tlp3) of *Campylobacter jejuni*. PLoS Pathog..

[77] Hasan M.A., Khan M.A., Sharmin T., Mazumder M.H.H., Chowdhury A.S. (2016). Identification of putative drug targets in Vancomycin-resistant *Staphylococcus aureus* (VRSA) using computer aided protein data analysis. Gene.

[78] Hobohm U., Scharf M., Schneider R., Sander C. (1992). Selection of representative protein data sets. Protein Sci..

[79] Wang F., Xiao J., Pan L., Yang M., Zhang G., Jin S., Yu J. (2008). A systematic survey of mini-proteins in bacteria and archaea. PLoS ONE.

[80] Gupta S.K., Sarita S., Gupta M.K., Pant K.K., Seth P.K. (2010). Definition of potential targets in *Mycoplasma pneumoniae* through subtractive genome analysis. J. Antivir. Antiretrovir..

[81] Haag N.L., Velk K.K., Wu C. (2012). In silico identification of drug targets in methicillin/multidrug-resistant *Staphylococcus aureus*. Int. J. Adv. Life Sci..

[82] Uddin R., Saeed K. (2014). Identification and characterization of potential drug targets by subtractive genome analyses of methicillin resistant *Staphylococcus aureus*. Comput. Biol. Chem..

[83] Zhang C., Xia Y. (2009). Identification of genes differentially expressed in vivo by Metarhizium anisopliae in the hemolymph of *Locusta migratoria* using suppression-subtractive hybridization. Curr. Genet..

[84] Restrepo-Montoya D., Vizcaíno C., Niño L.F., Ocampo M., Patarroyo M.E., Patarroyo M.A. (2009). Validating subcellular localization prediction tools with mycobacterial proteins. BMC Bioinform..

[85] Duffield M., Cooper I., McAlister E., Bayliss M., Ford D., Oyston P. (2010). Predicting conserved essential genes in bacteria: In silico identification of putative drug targets. Mol. Biosyst..

[86] Novick R.P., Geisinger E. (2008). Quorum sensing in staphylococci. Annu. Rev. Genet..

[87] Ng W.-L., Bassler B.L. (2009). Bacterial quorum-sensing network architectures. Annu. Rev. Genet..

[88] Williams P., Cámara M. (2009). Quorum sensing and environmental adaptation in *Pseudomonas aeruginosa*: A tale of regulatory networks and multifunctional signal molecules. Cur. Opin. Microbiol..

[89] Barrett J.F., Hoch J.A. (1998). Two-component signal transduction as a target for microbial anti-infective therapy. Antimicrob. Agents Chemother..

[90] Cai X.-H., Zhang Q., Shi S.-Y., Ding D.-F. (2005). Searching for potential drug targets in two-component and phosphorelay signal-transduction systems using three-dimensional cluster analysis. Acta Biochim. Biophys. Sin. (Shanghai).

[91] Vollmer W., Bertsche U. (2008). Murein (peptidoglycan) structure, architecture and biosynthesis in *Escherichia coli*. Biochim. Biophys. Acta.

[92] de Pedro M.A., Cava F. (2015). Structural constraints and dynamics of bacterial cell wall architecture. Front. Microbiol..

[93] Basavannacharya C., Robertson G., Munshi T., Keep N.H., Bhakta S. (2010). ATP-dependent MurE ligase in *Mycobacterium tuberculosis*: Biochemical and structural characterisation. Tuberculosis.

[94] Natale P., Brüser T., Driessen A.J. (2008). Sec-and Tat-mediated protein secretion across the bacterial cytoplasmic membrane—Distinct translocases and mechanisms. Biochim. Biophys. Acta.

[95] Ochsner U.A., Snyder A., Vasil A.I., Vasil M.L. (2002). Effects of the twin-arginine translocase on secretion of virulence factors, stress response, and pathogenesis. Proc. Natl. Acad. Sci. USA.

[96] Pradel N., Ye C., Livrelli V., Xu J., Joly B., Wu L.-F. (2003). Contribution of the twin arginine translocation system to the virulence of enterohemorrhagic *Escherichia coli* O157: H7. Infect. Immun..

[97] Lavander M., Ericsson S.K., Bröms J.E., Forsberg A. (2006). The twin arginine translocation system is essential for virulence of *Yersinia pseudotuberculosis*. Infect. Immun..

[98] Young G.M., Schmiel D.H., Miller V.L. (1999). A new pathway for the secretion of virulence factors by bacteria: The flagellar export apparatus functions as a protein-secretion system. Proc. Natl. Acad. Sci. USA.

[99] Mühlen S., Dersch P. (2015). Anti-virulence strategies to target bacterial infections. How to Overcome the Antibiotic Crisis.

[100] Xing X., Bi S., Fan X., Jin M., Liu W., Wang B. (2019). Intranasal Vaccination with Multiple Virulence Factors Promotes Mucosal Clearance of *Streptococcus suis* Across Serotypes and Protects Against Meningitis in Mice. J. Infect. Dis..

[101] Guarner F., Malagelada J.-R. (2003). Gut flora in health and disease. Lancet.

[102] Sears C.L. (2005). A dynamic partnership: Celebrating our gut flora. Anaerobe.

[103] Senes A., Ubarretxena-Belandia I., Engelman D.M. (2001). The Cα—H⋯ O hydrogen bond: A determinant of stability and specificity in transmembrane helix interactions. Proc. Natl. Acad. Sci. USA.

[104] Adamian L., Liang J. (2002). Interhelical hydrogen bonds and spatial motifs in membrane proteins: Polar clamps and serine zippers. Proteins.

[105] Curran A.R., Engelman D.M. (2003). Sequence motifs, polar interactions and conformational changes in helical membrane proteins. Curr. Opin. Struct. Biol..

[106] Xiong J. (2006). Essential Bioinformatics.

[107] Chu K.H., Wong S.H., Leung P.S. (2000). Tropomyosin is the major mollusk allergen: Reverse transcriptase polymerase chain reaction, expression and IgE reactivity. Mar. Biotechnol..

[108] Nisbet A.J., Huntley J.F. (2006). Progress and opportunities in the development of vaccines against mites, fleas and myiasis-causing flies of veterinary importance. Parasite Immunol..

[109] Rappuoli R. (2000). Reverse vaccinology. Curr. Opin. Struct. Biol..

[110] Bhavsar A.P., Guttman J.A., Finlay B.B. (2007). Manipulation of host-cell pathways by bacterial pathogens. Nature.

[111] Stavrinides J., McCann H.C., Guttman D.S. (2007). Host–pathogen interplay and the evolution of bacterial effectors. Cell. Microbiol..

[112] Simeone R., Bottai D., Brosch R. (2009). ESX/type VII secretion systems and their role in host–pathogen interaction. Curr. Opin. Struct. Biol..

[113] Parizi L.F., Ali A., Tirloni L., Oldiges D.P., Sabadin G.A., Coutinho M.L., Seixas A., Logullo C., Termignoni C., Da Silva Vaz I. (2018). Peptidase inhibitors in tick physiology. Med. Vet. Entomol..

[114] Ali A., Khan S., Ali I., Karim S., da Silva Vaz I., Termignoni C. (2015). Probing the functional role of tick metalloproteases. Physiol. Entomol..

[115] Durrant J.D., McCammon J.A. (2011). Molecular dynamics simulations and drug discovery. BMC Biol..

[116] Lee C., Kim M.I., Park J., Jeon B.-Y., Yoon S., Hong M. (2017). Crystal structure of the flagellar chaperone FliS from *Bacillus cereus* and an invariant proline critical for FliS dimerization and flagellin recognition. Biochem. Biophys. Res. Commun..

