# Correction: Ali, A., et al. Modeling Novel Putative Drugs and Vaccine Candidates against Tick-Borne Pathogens: A Subtractive Proteomics Approach. *Vet. Sci.* 2020, *7*, 129

**DOI:** 10.3390/vetsci7040158

**Published:** 2020-10-20

**Authors:** Abid Ali, Shabir Ahmad, Abdul Wadood, Ashfaq U. Rehman, Hafsa Zahid, Muhammad Qayash Khan, Javed Nawab, Zia Ur Rahman, Abdulaziz S. Alouffi

**Affiliations:** 1 Department of Zoology, Abdul Wali Khan University Mardan, Khyber Pakhtunkhwa 23200, Pakistan; uop_ali@yahoo.com (A.A.); shabirjan427@gmail.com (S.A.); hafsa.zahid@awkum.edu.pk (H.Z.); qayashkhan@awkum.edu.pk (M.Q.K.); 2 Department of Biochemistry, Abdul Wali Khan University Mardan, Khyber Pakhtunkhwa 23200, Pakistan; awadood@awkum.edu.pk (A.W.); serendifity_rehman@outlook.com (A.U.R.); 3 State Key Laboratory of Microbial Metabolism, Department of Bioinformatics and Biostatistics, National Experimental Teaching Center for Life Sciences and Biotechnology, College of Life Sciences and Biotechnology, Shanghai Jiao Tong University, Shanghai 200240, China; 4 Department of Environmental Sciences, Abdul Wali Khan University Mardan, Khyber Pakhtunkhwa 23200, Pakistan; javednawab11@yahoo.com; 5 Department of Microbiology, Abdul Wali Khan University Mardan, Khyber Pakhtunkhwa 23200, Pakistan; zrahman@awkum.edu.pk; 6 King Abdulaziz City for Science and Technology, Riyadh 12354, Saudi Arabia

The authors wish to make the following corrections to this paper [1].

Figure 4 should be replaced due to mislabeling. The correct Figure 4 can be found below.

The authors would like to apologize for any inconvenience caused to the readers by these changes.

## Figures and Tables

**Figure 4:**
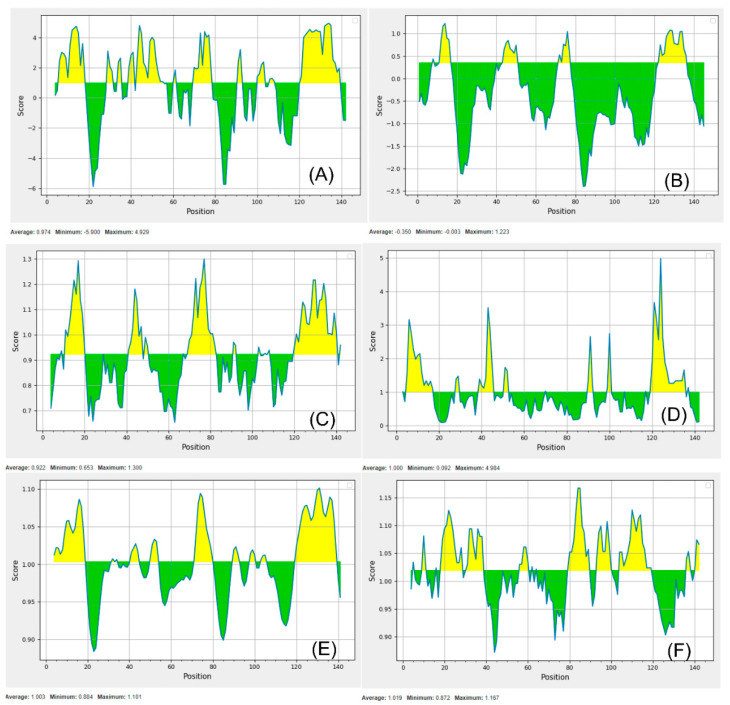
B-cell epitope in the FLiS protein based on Parker hydrophilicity prediction (**A**). Bepipred linear epitope (**B**). Chou and Fasman β-turn prediction (**C**). Emini surface accessibility prediction (**D**). Karplus and Schulz flexibility prediction (**E**). Kolaskar and Tongaonkar antigenicity (**F**). The x axis and y axis represent the sequence position and corresponding antigenic properties score, respectively. The threshold level was set as a default parameter of the server. The regions shown in yellow color above the threshold value were predicted as the B-cell epitope.
